# Supplementary material for: Highly Species-Specific Foliar Metabolomes of Diverse Woody Species and Relationships with the Leaf Economics Spectrum
Source: Cells. 2021 Mar 13;10(3):644. doi: 10.3390/cells10030644 (PMC7999030; doi:10.3390/cells10030644)
Supplement: Supplementary file 1 [file cells-10-00644-s001.pdf]

cells

MDPI

Supplement

Highly Species-Specific Foliar Metabolomes of Diverse Woody Species and Relationships with the Leaf Economics Spectrum

Rabea Schweiger, Eva Castells, Luca da Sois, Jordi Martínez-Vilalta and Caroline Müller

Supplementary Table S1: Retention indices (RI) and mass to charge ratios (m/z) of the identified metabolites. RI were calculated from the GC-FID measurements while the m/z values, shown as *m/z (intensity)*, were derived from the GC-MS measurements. All the metabolites were also identified via comparison with reference standards.

Metabolite	RI	Mass to charge ratio (m/z)
**Sugars**		
fructose	1828/1838	73 (100), 307 (81.6), 217 (80.7), 103 (78.9), 147 (39.6), 308 (22.5), 277 (15.6), 133 (14.1), 205 (10.2), 364 (10.2)
glucose	1853/1873	319 (100), 73 (74.9), 205 (57.8), 147 (40.0), 160 (31.0), 320 (29.5), 321 (14.5), 206 (14.4), 103 (11.7), 204 (8.5)
galactose	1846/1869	319 (100), 205 (74.6), 147 (37.8), 320 (31.7), 160 (22.4), 206 (18.3), 321 (14.7), 204 (9.9), 75 (9.5), 229 (8.7)
sucrose	2607	
**Organic acids**		
oxalic acid	1134	147 (100), 73 (67.5), 148 (15.3), 190 (8.2), 149 (7.5), 74 (6.0), 66 (5.1), 72 (4.8), 219 (4.8), 133 (4.0)
citric acid	1779	273 (100), 73 (67.8), 147 (59.3), 363 (26.9), 347 (24.8), 375 (23.5), 465 (15.0), 75 (11.1), 275 (9.8), 149 (9.5)
**Polyalcohols**		
myo-inositol	2051	305 (100), 217 (82.4), 73 (64.9), 318 (57.4), 147 (51.0), 191 (39.3), 319 (24.3), 265 (19.8), (16.9), 432 (12.8)

Supplementary Figure S1: NMDS-contour line plots for C per leaf area and N per area.

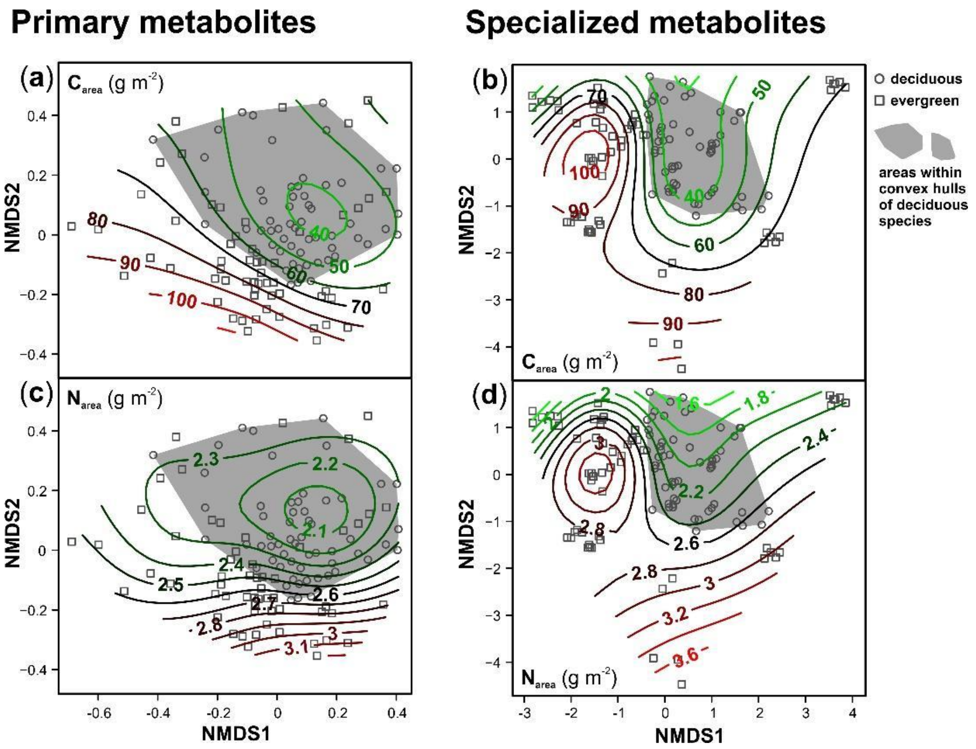

Figure S1. Simplified non-metric multidimensional scaling plots of relative concentrations of metabolic features of the primary (left) and specialized (right) metabolism detected in leaves of 20 plant species (deciduous: circles; evergreen: squares). The deciduous species are highlighted by gray areas that are framed by convex hulls (closed curves surrounding all data points with minimum perimeter) for this group. Contour lines (green: low values; red: high) representing surface fits of generalized additive models for: (**a**, **b**) carbon (C) per leaf area (C_{area}); (**c**, **d**) nitrogen (N) per area (N_{area}).
